# Association between impulse oscillometry Z-scores and asthma control and exacerbation risk in a tertiary severe asthma clinic

**DOI:** 10.3389/falgy.2026.1741154

**Published:** 2026-02-12

**Authors:** Li Ping Chung, Dylan Beinart, Emily S. Y. Goh, Gregory G. King

**Affiliations:** 1 Severe Airways Disease Clinic, Fiona Stanley Hospital, Perth, WA, Australia; 2 Department of Respiratory Medicine, Royal North Shore Hospital, Sydney, NSW, Australia; 3 Airway Physiology and Imaging Group, The Woolcock Institute of Medical Research, Sydney, NSW, Australia

**Keywords:** asthma, asthma control test, asthma exacerbation, impulse oscillometry, lung function test, respiratory oscillometry, small airways dysfunction

## Abstract

**Introduction:**

Respiratory oscillometry is a sensitive tool for assessing small airways dysfunction. However, limited evidence on cutoff values for interpretation remains a barrier to its clinical use. The aim of this study was to determine whether the presence and severity of abnormalities, defined by Z-scores for oscillometric parameters, are associated with asthma symptoms and exacerbation risk.

**Methods:**

We retrospectively reviewed the medical records of all patients with asthma managed in a severe asthma clinic between 2019 and 2022 who underwent routine oscillometry. Z-scores for oscillometric parameters were analyzed as continuous and categorical variables to assess their associations with asthma control and exacerbation risk.

**Results:**

When analyzed as categorical variables, Z-score-defined severity thresholds for resistance (R_5_), reactance (X_5_), and the area under the reactance curve (A_X_) were associated with levels of asthma control (as measured by the ACQ5). When analyzed as continuous variables, Z-scores were also correlated with worst asthma control (as assessed by both ACQ5 and the asthma control test) (*P* < 0.005). These correlations remained significant after adjustment for spirometric indices, FeNO, and treatment changes. Elevated Z-scores (>1.64) for R_5_ were associated with a higher risk of exacerbations (OR 2.70, 95% CI 1.27–5.17, *P* = 0.009). The risk of exacerbation increased with the severity of airway obstruction. Similar trends were observed for A_X_ and X_5_; however, these associations did not reach statistical significance.

**Discussion:**

The presence and severity of airway obstruction, as defined by R_5_ Z-scores, predict poorer asthma control and an increased risk of exacerbations. Similar associations with asthma control were also observed for X_5_ and A_X_ Z-scores. Clinicians should use Z-scores over other cutoffs to aid interpretation.

## Introduction

Respiratory oscillometry is a simple, non-invasive, and effort-independent lung function test that overlays oscillatory pressure waves onto normal tidal breathing to measure the mechanical properties of the airways and lung parenchyma (1). Compared with spirometry, respiratory oscillometry is more sensitive for assessing the peripheral or small airways, especially in patients with chronic respiratory symptoms and preserved pulmonary function (2–4). Small airways dysfunction is highly prevalent in adults with asthma and is associated with the degree of airflow obstruction, symptom burden, and risk of exacerbations (5–7).

Introducing oscillometric assessment of small airways function into routine clinical practice provides clinicians with a more comprehensive understanding of airway physiology, enabling better assessment of disease activity and the risk of asthma exacerbations. Respiratory oscillometry therefore provides further information to spirometry when assessing current disease control and predicting clinical outcomes (4, 8). For example, patients with asthma who exhibit higher respiratory resistance, indicating worse small airways dysfunction, have been shown to achieve a better response to extra-fine particle inhaled therapy compared with non-extra-fine therapy (9, 10).

A recently published international Delphi study on the interpretation of respiratory oscillometry in adults with asthma or chronic obstructive pulmonary disease (COPD) reported that clinicians who routinely use oscillometry in clinical practice focus on a small number of metrics to guide interpretation, specifically resistance at 5 Hz (R_5_), frequency dependence of resistance (R_5_–R_20_ or R_5_–R_19_), reactance at 5 Hz (X_5_), and area under the reactance curve (A_X_) (11). This expert group agreed that respiratory oscillometry is clinically useful for identifying and grading the severity of lung function impairment, as well as for assessing clinically meaningful changes in lung function over time. The group recommended the use of Z-scores to define abnormal lung function, with cutoffs of >1.64 for R_5_ and A_X_ and <−1.64 for X_5_. Because X_5_ values are usually negative, more negative values indicate greater impairment in lung function. The severity of abnormal lung function was further defined according to the criteria outlined in Table 1.

**Table 1:** Grading the severity of abnormal lung function.

Severity of abnormal lung function	Oscillometry parameter	
	R_5_, R_20_, A_X_, F_res_	X_5_
Normal (none)	Z-score ≤ 1.64	Z-score ≥ −1.64
Mild	Z-score > 1.64 and ≤ 2.5	Z-score < −1.64 and ≥ −2.5
Moderate	Z-score > 2.5 and ≤ 4	Z-score < −2.5 and ≥ −4
Severe	Z-score > 4	Z-score < −4

Although Z-score cutoffs for impedance parameters provide a statistically robust framework for defining the severity of abnormality, empiric data are required to demonstrate their clinical relevance and validity (11). This evidence gap represents a barrier to some clinicians using this lung function test in clinical practice.

We have previously demonstrated that, among patients with asthma attending our tertiary asthma clinic, R_5_–R_20_, X_5_, A_X_, and resonant frequency (F_res_) are correlated with asthma symptom burden, with the strongest association observed for R_5_–R_20_. Both A_X_ and R_5_–R_20_ were associated with an increased risk of asthma exacerbations (5). However, this previous analysis was predominantly based on abnormal lung function defined by absolute value cutoffs commonly reported in published studies, rather than Z-scores. As the use of absolute values to define abnormal oscillometry findings was not endorsed by the Delphi study (11) and is subject to inherent limitations (12), the aim of this study was to determine whether the presence and severity of abnormal lung function, defined using Z-scores for oscillometric parameters, are associated with asthma symptoms and exacerbation risk.

## Methods

This was a single-center, retrospective study of patients with asthma referred to a tertiary respiratory clinic who underwent oscillometry as part of their routine assessment between January 2019 and December 2022. The study was approved by the Human Research Ethics Committee and Research Governance Unit of Fiona Stanley Hospital (RGS5611).

The methods of this study have been previously published (5) and are briefly described here. Eligible patients had a respiratory specialist-confirmed diagnosis of asthma and had completed spirometry and oscillometry, specifically impulse oscillometry (IOS), as part of standard lung function testing. Patients were excluded if they did not have at least one documented IOS measurement performed at our tertiary clinic.

The relevant information was extracted from the medical records of all eligible patients corresponding to their clinic visit at which lung function testing was performed. Collected information included standard demographic data, asthma symptom scores [e.g., asthma control questionnaire (ACQ5) or asthma control test (ACT)], frequency of asthma exacerbations in the 12 months before and after the IOS test, asthma medications, asthma severity (based on GINA criteria), and IOS results. Asthma exacerbations were defined as any worsening of asthma symptoms that required treatment with antibiotics and/or oral corticosteroids or resulted in an unscheduled visit to an accident and emergency department, a hospital, or a general practitioner (13, 14). All exacerbations that occurred were included in the analysis.

Oscillometry was performed in accordance with the manufacturer's recommendations using an impulse oscillometry device (Masterscreen IOS, Jaeger, Germany). Typically, oscillometry was performed on the same day as the respiratory specialist review or within 48 h prior.

Prebronchodilator IOS parameters including R_5_, R_20_, A_X_, F_res_, and X_5_ were analyzed. Normative values for oscillometric parameters were calculated based on data published by Oostveen (15). Z-scores for R_5_, R_20_, X_5_, A_X_, and F_res_ were evaluated as continuous and categorical variables to assess their associations with asthma control and exacerbation risk. Other IOS parameters, such as R_5_–R_20_, were not included in this analysis because normative data for these metrics are unavailable; hence, Z-scores could not be calculated. For categorical variables, abnormal lung function was defined as a Z-score > 1.64 for R_5_, R_20_, A_X_, and F_res_ and a Z-score < −1.64 for X_5_. The severity of dysfunction was defined a mild, moderate, or severe based on the Z-scores listed in Table 1.

### Statistical analysis

Correlations between Z-scores of oscillometry parameters and asthma symptom scores (ACQ5 and ACT), evaluated as continuous variables using ANOVA regression analysis, are reported as Pearson correlation coefficients. The variables were confirmed to be normally distributed. For categorical analyses, mean ACQ5 and ACT scores for mild, moderate, and severe Z-score categories were compared with those of patients with normal Z-scores using multiple *t*-tests. Exacerbation risk across Z-score categories was compared using chi-square analysis. Multiple regression and logistic analyses were performed to adjust for potential confounders, including spirometric airflow obstruction (FEV_1_% predicted, FEV_1_/FVC < 0.70), FeNO, and treatment changes within 12 months after IOS testing.

No power calculations were performed, as this was a retrospective study that included all eligible patients. Statistical analyses were performed using Jamovi, version 2.2.5.

When relevant data were not documented in the patient record, patients were excluded from that analysis. For example, the absence of information about exacerbation history was not assumed to indicate that no exacerbation had occurred.

## Results

A total of 149 patients were included in this retrospective study. Based on GINA criteria, 69% of patients were classified as having severe asthma (14). Nearly 90% of patients were receiving inhaled corticosteroid-based combination therapy, with equal proportions treated with inhaled corticosteroids (ICS) plus long-acting beta-2 agonists (LABA) and ICS/LABA plus long-acting muscarinic antagonists (LAMA). Based on the FEV_1_/FVC ratio, 64% of patients had obstructive airflow. These clinical characteristics are consistent with the typical patient cohort referred to a tertiary asthma clinic. Demographic and clinical characteristics are summarized in Table 2.

**Table 2:** Patient demographics and clinical characteristics, including the prevalence of abnormal lung function as assessed by oscillometry.

Demographics (*N* = 149)	*N* (%) *Median (IQR)
Age (years)*	49.33 (34.81–62.19)
Male/female (%)	55/94 (37.2/63.0)
Ethnicity—Caucasian/other (%)	122/27 (81.9/18.2)
BMI (kg/m^2^)*	30.09 (24.77–35.36)
Smoking status (%)	
Current	7 (4.7)
Former	57 (38.2)
Never	85 (57.4)
Exacerbation in the preceding or proceeding 12 months (%)	
Yes	81 (54.4)
No	56 (37.8)
Not documented (unknown)	12 (8.1)
Inhaled therapy	*N* (%)
None	8 (5.4)
ICS monotherapy	7 (4.7)
ICS/LABA	66 (44.6)
LAMA/LABA (no ICS)	1 (0.7)
Single-inhaler triple therapy (ICS/LABA/LAMA)	11 (6.8)
Triple therapy (ICS/LABA/LAMA) using multiple inhalers	67 (44.9)
Systemic therapy	*N* (%)
Montelukast	28 (18.9)
Oral corticosteroids (maintenance)	21 (14.2)
Biologic (monoclonal antibody)	25 (16.9)
Pulmonary function tests	Median (IQR)
FEV_1_% predicted (%)	73.3 (57.1–85.0)
FVC % predicted (%)	92.9 (78.5–100.8)
FEV_1_/FVC ratio (%)	62.5 (57.1–85.9)
FeNO (ppb)	31.5 (20.8–60.0)
R_5Hz_ [kPa/(L/s)]	0.54 (0.40–0.75)
R_5Hz_ % predicted (%)	173.15 (133.98–229.38)
R_20Hz_ [kPa/(L/s)]	0.38 (0.30–0.46)
R_20Hz_ % predicted (%)	136.40 (117.08–166.93)
R_5_–R_20_ [kPa/(L/s)]	0.13 (0.08–0.28)
ΔR_5_–R_20_% (%)	35.26 (22.99–59.68)
A_X_ (kPa/L)	0.66 (0.27–2.10)
BF (L/min)	12.98 (10.68–16.49)
F_res_ (Hz)	15.00 (10.75–20.92)
X_5_ [kPa/(L/s)]	−0.17 (−0.28–−0.11)
X_5_% predicted (%)	177.00 (115.45–248.20)
Prevalence of abnormal lung function (based on Z-scores, *N* = 149)	*N* (%)
R_5_ (Z-score > 1.64)	105 (70.5)
R_20_ (Z-score > 1.64)	41 (27.5)
A_X_ (Z-score > 1.64)	44 (29.5)
X_5_ (Z-score < −1.64)	67 (45.0)
F_res_ (Z-score > 1.64)	35 (23.4)

Of the 149 patients, 101 (67.8%) had a change in treatment within 12 months after IOS testing. These changes include commencement or switching of a biologic agent (*N* = 39), ICS dose escalation and/or change to a fine or extra-fine particle ICS formulation (*N* = 26), or commencement of a LAMA or montelukast (*N* = 12). In addition, 16 patients underwent treatment “step-down” after optimization of inhaler technique and adherence to original treatments.

The prevalence of abnormal resistance (R_5_), defined by Z-scores > 1.64, was high at 70.4%. In contrast, the prevalence of small airways dysfunction was lower, occurring in 40.5% as defined by X_5_ Z-score < −1.64 and in 29.5% as defined by A_X_ Z-scores > 1.64. Abnormal lung function for R_5_, X_5_, and A_X_ was associated with poorer symptom control, as assessed by ACQ5 and ACT, compared with patients who had normal oscillometric findings. No significant associations were observed between abnormal F_res_ and R_20_ and asthma control (Table 3).

**Table 3:** Differences in asthma control between patients with normal vs. abnormal small airways function.

IOS parameter	*N*	Asthma control			
		Mean ACQ5	P	Mean ACT	P
R_5_ normal (Z-score ≤ 1.64)	44	1.20	<0.0001	19.54	=0.001
R_5_ abnormal (Z-score > 1.64)	105	2.15		16.59	
X_5_ normal (Z-score ≥ −1.64)	82	1.20	<0.0001	18.39	=0.011
X_5_ abnormal (Z-score < −1.64)	67	2.18		16.19	
A_X_ normal (Z-score ≤ 1.64)	105	1.64	=0.0002	17.84	=0.03
A_X_ abnormal (Z-score > 1.64)	44	2.40		13.67	
R_20_ normal (Z-score ≤ 1.64)	108	1.62	=0.241	16.88	=0.13
R_20_ abnormal (Z-score > 1.64)	41	1.98		18.76	
F_res_ normal (Z-score ≤ 1.64)	114	1.77	=0.059	19.44	=0.26
F_res_ abnormal (Z-score > 1.64)	35	2.26		16.51	

Statistical test: Multiple *t*-tests.

**Figure 1:**
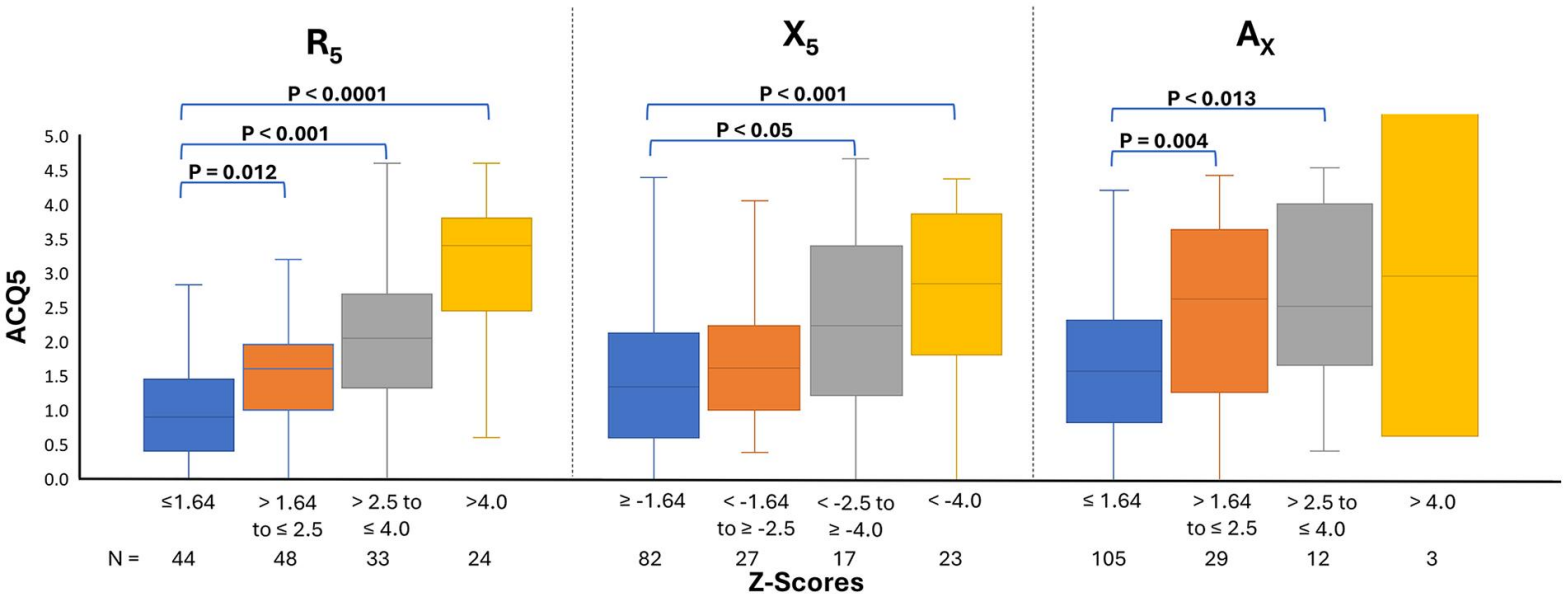
Relationship between the severity of abnormal lung function and asthma control as measured by ACQ5. Statistical test: Multiple *t*-tests.

When analyzed as categorical variables, Z-score-defined severity of lung function impairment for R_5_, X_5_, and A_X_ was significantly associated with the level of asthma control. Increasing severity of lung function impairment corresponded to poorer asthma control. This relationship was strongest when asthma control was measured using ACQ5 (Figure 1). The strength of these associations with ACT was similar for R_5_ but weaker for X_5_ and A_X_ compared with ACQ5 (Supplementary Table S1).

**Figure 2:**
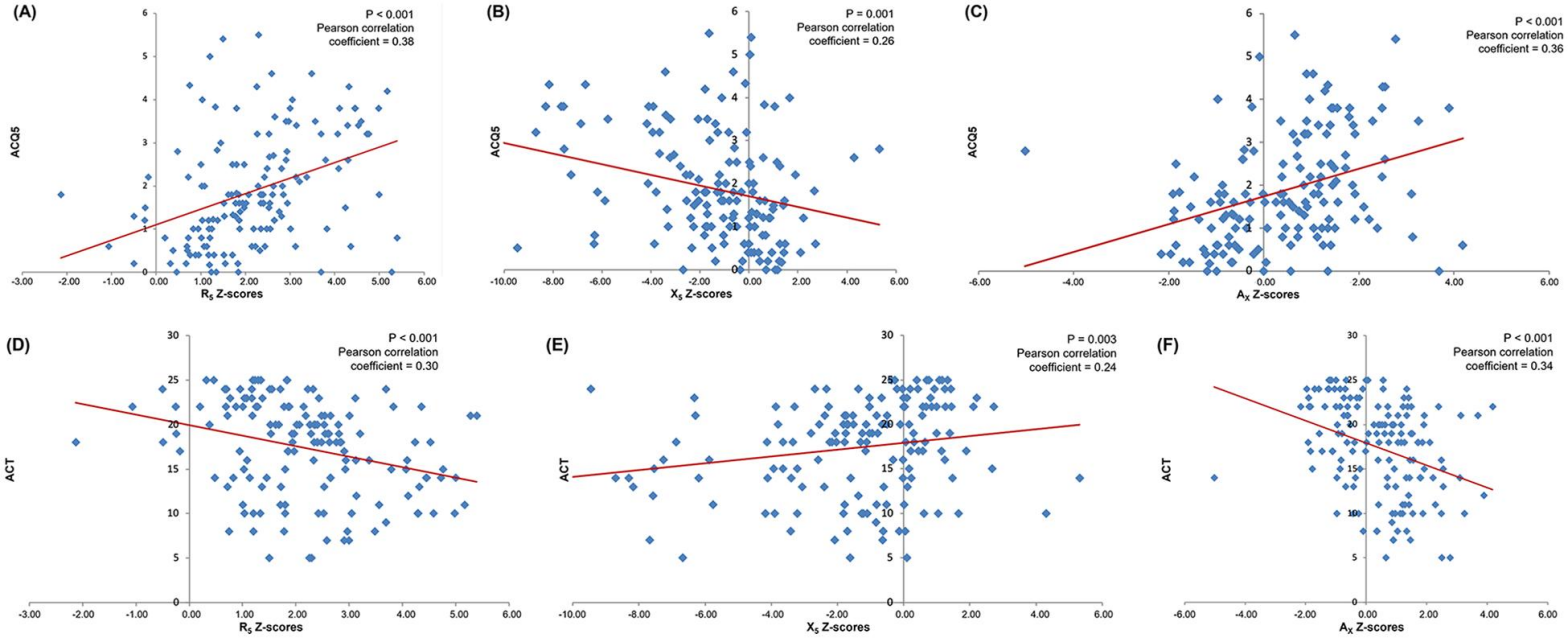
Correlations between IOS parameter Z-scores and asthma control as measured by ACQ5 [ **(A)** R_5_, **(B)** X_5_, and **(C)** A_X_] and the ACT [ **(D)** R_5_, **(E)** X_5_, and **(F)** A_X_]. Statistical test: ANOVA regression analysis.

Z-scores for R_5_, X_5_, and A_X_ were all significantly correlated with asthma control scores (ACQ5: R_5_ *r* = 0.38, *P* < 0.001, X_5_ *r* = 0.26, *P* = 0.001, A_X_ *r* = 0.36, *P* < 0.001; ACT: R_5_ *r* = 0.30, *P* < 0.001, X_5_ *r* = 0.24, *P* = 0.003, A_X_ *r* = 0.34, *P* < 0.001). The worse the Z-score, the poorer the asthma control, as assessed by both ACQ5 and ACT (Figures 2A–F).

On multivariate analysis, spirometric indices (FEV_1_% predicted) and FeNO were correlated with ACQ5 (*P* = 0.01 and *P* = 0.009, respectively), while FEV_1_% predicted and FEV_1_/FVC were correlated with ACT (*r* = 0.34, *P* = 0.001 and *r* = 0.28, *P* = 0.02, respectively). The associations between asthma symptom control and Z-scores for R_5_ and A_X_ remained significant after adjusting for spirometry, FeNO, and treatment changes (ACQ5 R_5_ *r* = 0.30, *P* = 0.005; ACT R_5_ *r* = 0.26, *P* = 0.05; and ACT A_X_ *r* = 0.29, *P* = 0.04).

**Figure 3:**
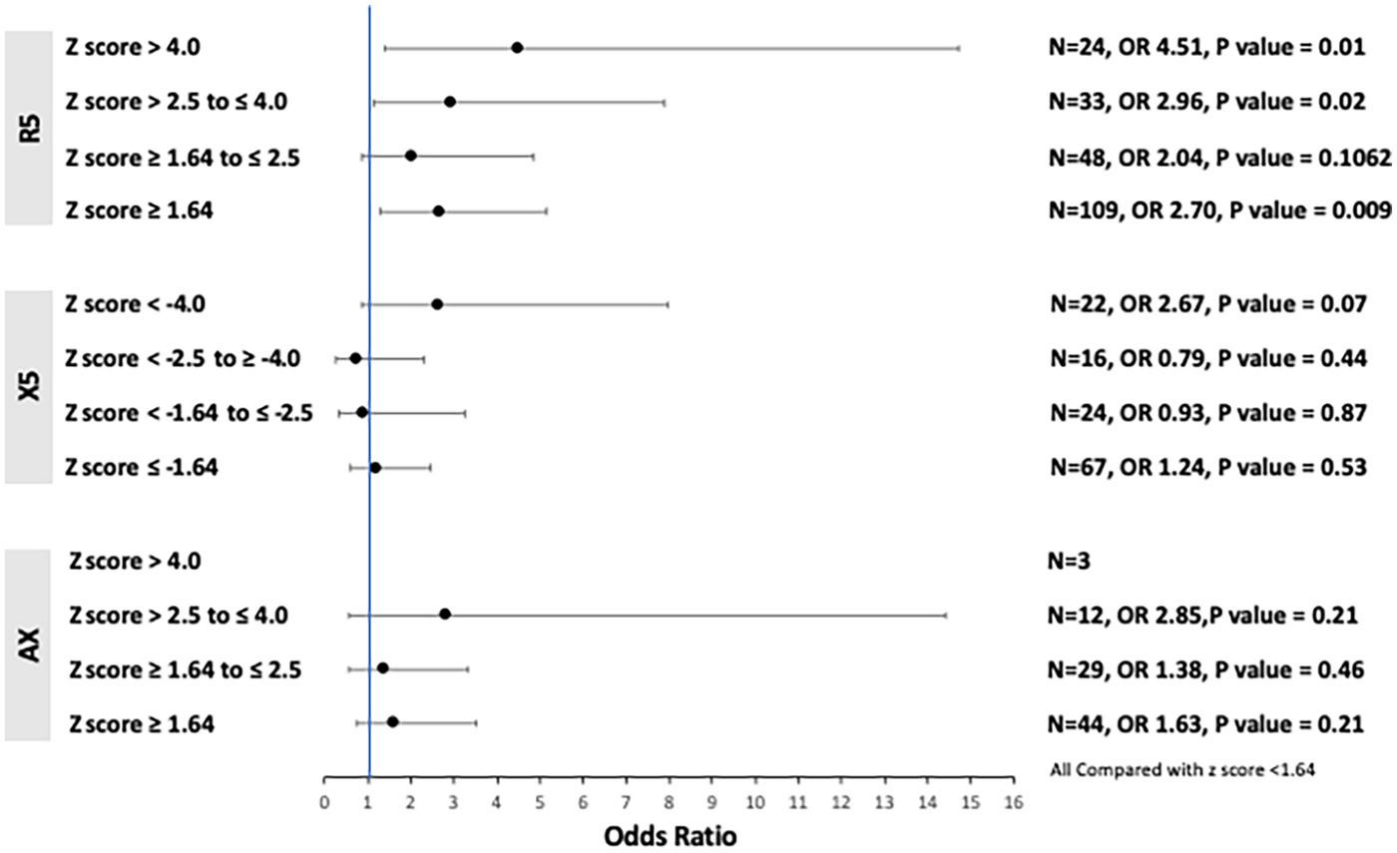
Relationships between impaired R_5_, X_5_, A_X_, and asthma exacerbations. Statistical test: Chi-square analysis.

Eighty-one patients (54%) experienced at least one documented moderate-to-severe asthma exacerbation in the 12 months before or after the sentinel date. Impaired resistance (R_5_) was associated with a significantly increased risk of exacerbation compared with patients with normal R_5_ (OR 2.70, 95% CI 1.27–5.17, *P* = 0.009). The risk of exacerbation increased with greater severity of airway obstruction (abnormal R_5_) (Figure 3). Impaired reactance, as reflected by both X_5_ and A_X_, demonstrated trends toward an elevated risk of exacerbations compared with patients who had normal reactance parameters (X_5_: OR 1.24, 95% CI 0.63–2.47, *P* = 0.53; A_X_: OR 1.63, 95% CI 0.75–3.53, *P* = 0.21). Similarly, there were non-significant trends suggesting that the risk of exacerbations increased with greater severity of impaired reactance. Similar to asthma control, exacerbation risk was not associated with abnormal R_20_ or F_res_ (Table 3). The association between R_5_ and the risk of exacerbations was not significant on multivariate analysis.

## Discussion

We have shown, in a retrospective study conducted within a tertiary referral clinic for severe asthma, that greater impairment in oscillometric parameters is associated with worse symptoms, i.e., asthma control, and with more frequent severe asthma exacerbations. The relationship between abnormal oscillometry based on Z-scores and poorer asthma control was strengthened by multivariate analysis and is consistent with the *post-hoc* analysis of the ATLANTIS study, in which small airways dysfunction was identified by A_X_, X_5,_ and R_5_–R_20_ (7). These findings support key recommendations from the recent Delphi study to use Z-scores for defining abnormal lung function as assessed by oscillometry (11).

Significant associations between R_5_, X_5_, and A_X_ Z-scores and asthma symptoms used to define control are consistent with our previous analysis, in which abnormal lung function was determined using absolute cutoffs most commonly reported in other studies (5). Similarly, the ATLANTIS study demonstrated that higher absolute values of R_5_ and A_X_ were associated with poorer asthma control (as reflected by lower ACT scores) (6). In addition, Abdo et al. (16) reported significant associations between A_X_ and asthma control using both the ACT and ACQ7.

In terms of grading the severity of abnormal oscillometry, Liang developed a severity grading system based on categories predicted by FEV_1_ Z-scores among patients with asthma (17). For R_5_, the classifications of mild, moderate, and severe abnormality closely matched those used in clinical practice (11) and those applied in our study (Table 1). For X_5_, moderate impairment was calculated as Z-scores between <−4.5 and ≥−8.5, which differs from the thresholds used in our study (<−2.5 to ≥−4), based on consensus recommendations (11). Liang acknowledged that a major limitation of their methodology was the limited consistency between oscillometry and spirometry and consequently proposed that cutoffs for oscillometry parameters should be guided by clinical practice, as was done in our study (11). However, these discrepancies highlight the need for additional research to further define the severity of abnormal oscillometry.

In our study, the associations between oscillometric parameters and exacerbation risk were strongest for R_5_, a measure of airway caliber across the entire airway tree (1). The associations between X_5_ and A_X_, indicators of small airways dysfunction, and exacerbation risk were weaker. This finding that resistance-based parameters are stronger predictors than reactance is also consistent with the *post-hoc* analysis of the ATLANTIS study, which focused on patients with mild asthma. In that study, the investigators also used IOS, although Z-scores were derived from 100 healthy individuals included in the study. Thus, our findings indicate that Z-scores for defining severity and abnormality are relatively robust, at least between the two reference equations used (7).

We found no statistically significant associations between X_5_ and A_X_ and asthma exacerbations, whereas a previous analysis from the ATLANTIS study found that X_5_, A_X_, and R_5_–R_20_ were significantly correlated with asthma exacerbations and that a composite ordinal score based on these three parameters independently predicted the exacerbation risk (18). A retrospective study by Chan and Lipworth found that small airways dysfunction, defined by an A_X_ ≥ 1.0 kPa/L and R_5_–R_20_ ≥ 0.10 kPa/L/s, was significantly associated with asthma exacerbations (19). Similarly, Gao et al. (20) demonstrated that small airways dysfunction was an important pathological feature among patients with asthma exacerbations and that X_5_, A_X_, and R_5_–R_20_ were significantly correlated with asthma exacerbations. Measures of reactance, X_5_ and A_X_, reflect physiologically severe airway narrowing and greater heterogeneity of ventilation. Failure to demonstrate significant relationships with exacerbation risk may be attributable to fewer patients exhibiting moderate to severe impairment when defined using the applied Z-score cutoffs. Greater airway closure and heterogeneity, as measured by single-breath nitrogen washout, has been shown to be associated with an increased risk of exacerbations (21, 22). Whether resistance or reactance parameters relate to exacerbations may differ across populations and may be expected given the marked heterogeneity in underlying pathophysiology among patients.

One of the challenges for clinicians who are less familiar with respiratory oscillometry is the large number of oscillometric parameters and the resulting uncertainty about which ones to use in clinical practice. Similar to spirometry, where interpretation is predominantly based on three core parameters, namely, FEV_1_, forced vital capacity (FVC), and FEV_1_/FVC (12), the international Delphi study on the interpretation of respiratory oscillometry recommended using three oscillometry indices: R_5_, X_5_, and A_X_ (11). Hence, the findings of our study provide further insight and guidance to clinicians on the clinical significance of these parameters. Results from several studies suggest that respiratory oscillometry should be used in conjunction with spirometry (rather than as a replacement for it) (18, 23, 24), as their combination provides a more comprehensive assessment of lung physiology and clinical risks.

The main limitation of this study is its retrospective design and the exclusion of patients with inadequate data, including the presence or absence of exacerbations, as documented in their medical records. There are differences in measurements between oscillometry devices when tested in physical models or healthy participants (15, 25, 26). Differences in measurements between devices, as well as differences in normative values used to calculate Z-scores, may potentially affect the relationships between Z-scores and clinical outcomes (11). Therefore, our findings may not be generalizable to centers that use different oscillometry devices or prediction equations. Robust analysis differentiating the relationship between oscillometry measurements and previous or future exacerbations is limited by relatively few events and by the potential modifying effect of treatment escalations in two-thirds of the cohort in the year following IOS testing.

There are currently no well-established reference equations for the frequency dependence of resistance (R_5_–R_20_); hence, Z-score analysis of this measure of small airways dysfunction could not be performed. Normative data to derive Z-scores for R_5_–R_20_ are needed, as R_5_–R_20_ is a sensitive marker of small airways dysfunction (6), and impairement in R_5_–R_20_ has been associated with an increased risk of asthma exacerbation and poor symptom control (7, 16, 20), including a previously published analysis of this data set, in which abnormality was defined as R_5_–R_20_ > 0.07 kPa/(L/s) (5), as well as the ATLANTIS *post-hoc* analysis, where abnormal R_5_–R_20_ was defined as a Z-score > 1.645 (7).

One of the strengths of this study is that it was conducted among patients referred to a tertiary severe asthma clinic, a population that matches the patient cohort most likely to have access to respiratory oscillometry in the current real-world clinical setting. As the routine use of oscillometry expands, similar research should be performed in broader adult asthma patient populations managed in primary care. This would help to confirm the clinical utility of this lung function test in this clinical setting.

## Conclusion

The findings from our retrospective study provide real-world evidence supporting the use of R_5_ Z-scores to define abnormality in preference to other cutoff values, such as absolute values or percentage predicted. In addition, the arbitrary thresholds used to define the severity of abnormality appear to have some relevance in a severe asthma population managed at a tertiary referral clinic.

## Data Availability

The original contributions presented in the study are included in the article/Supplementary Material, further inquiries can be directed to the corresponding author.
