# Supplementary material for: Association between impulse oscillometry Z-scores and asthma control and exacerbation risk in a tertiary severe asthma clinic
Source: Front Allergy. 2026 Feb 12;7:1741154. doi: 10.3389/falgy.2026.1741154 (PMC12936017; doi:10.3389/falgy.2026.1741154)
Supplement: Supplementary file 1 [file Table1.docx]

The association between impulse oscillometry Z-scores and asthma control and exacerbation risk in a tertiary severe asthma clinic.

# Supplementary Data

**Supplementary Table 1: Relationship between the severity of abnormal lung function and asthma control as measured by ACT.**

| IOS parameter and severity of abnormality as defined by the Z-score | N | Asthma control  Mean ACT (SD) | P value* |
| --- | --- | --- | --- |
| **R_5_** | | | |
| None: Z-score ≤ 1.64 | 44 | 19.54 (5.02) | - |
| Mild: Z-score > 1.64 and ≤ 2.5 | 48 | 17.81 (5.53) | 0.012 |
| Moderate: Z-score > 2.5 and ≤ 4 | 33 | 17.10 (4.24) | < 0.001 |
| Severe: Z-score > 4 | 24 | 13.59 (4.15) | < 0.0001 |
| **X_5_** | | | |
| None: Z-score ≥ -1.64 | 82 | 18.39 (5.38) | - |
| Mild: Z-score < -1.64 and ≥ -2.5 | 27 | 17.01 (5.28) | 0.243 |
| Moderate: Z-score < -2.5 and ≥ -4 | 17 | 16.76 (4.68) | 0.025 |
| Severe: Z-score < -4 | 23 | 14.82 (4.75) | 0.004 |
| **A_X_** | | | |
| None: Z-score < 1.64 | 105 | 17.85 (5.41) | - |
| Mild: Z-score > 1.64 and ≤ 2.5 | 29 | 16.76 (4.70) | 0.32 |
| Moderate: Z-score > 2.5 and ≤ 4 | 12 | 14.50 (4.91) | 0.04 |
| Severe: Z-score > 4 | 3 | 13.67 (8.51) | - |

* Compared to no impairment (R5, AX Z-score < 1.64, X5 Z-score > -1.64)

Statistical test: Multiple T-tests
